# Application effect of Miller’s pyramid teaching method based on HeartCode BLS in basic life support training for nursing students

**DOI:** 10.3389/fmed.2026.1863479

**Published:** 2026-07-08

**Authors:** Wenxian Xu, Fanyi Cheng, Zhiping Li, Yajun Wang

**Affiliations:** 1 Department of Nursing, People’s Hospital of Quzhou, Quzhou, Zhejiang, China; 2 Department of Emergency Medicine, People’s Hospital of Quzhou, Quzhou, Zhejiang, China

**Keywords:** *HeartCode BLS*, *Miller’s pyramid*, *nursing students*, *randomized controlled trial*, *simulation-based education*

## Abstract

**Background:**

Basic life support (BLS) competence is a core professional skill for nursing students, yet conventional training often fails to achieve durable gains in knowledge, psychomotor performance, and clinical transfer. This study evaluated the effectiveness of a Miller’s pyramid-based HeartCode BLS teaching model compared with traditional instruction in undergraduate nursing students.

**Methods:**

In this randomized controlled trial, 229 eligible nursing students were enrolled and assigned to a control group (*n* = 115) or an observation group (*n* = 114). The observation group received a HeartCode BLS teaching model structured according to Miller’s pyramid, whereas the control group received conventional BLS teaching. Outcomes were assessed using the Kirkpatrick four-level evaluation framework, including post-training theoretical examination scores, 1-week skill performance, CPR quality indicators, 3-month high-fidelity simulation-based comprehensive application ability, and learner satisfaction. Between-group comparisons were conducted using chi-square tests, independent-samples *t*-tests, Mann–Whitney U tests, and repeated-measures ANOVA, as appropriate.

**Findings:**

Baseline characteristics and pre-training scores were comparable between groups. After training, the observation group achieved significantly higher scores than the control group in theoretical knowledge (74.29 ± 9.94 vs. 68.81 ± 9.33), CPR skills (85.92 ± 8.67 vs. 79.80 ± 7.56), and defibrillation skills (85.48 ± 8.63 vs. 78.86 ± 5.77; all *p* < 0.001). CPR quality indicators, including compression depth, compression rate, chest recoil, and bag-valve-mask ventilation, were also significantly better in the observation group (all *p* < 0.001). A greater proportion of students in the observation group maintained total compression interruption times below 30 s (57.0% vs. 25.2%; *p* < 0.001). Learner satisfaction was consistently higher across all domains (all *p* < 0.001). At 3 months, the observation group also outperformed the control group in emergency judgment and decision-making, emergency procedure execution, team communication and collaboration, and total simulation score (all *p* < 0.001).

**Interpretation:**

A Miller’s Pyramid-based HeartCode BLS teaching model was associated with significantly improved immediate learning outcomes, CPR quality, learner satisfaction, and mid-term integrated clinical performance among nursing students. This theory-informed, feedback-enabled instructional strategy may offer a scalable and effective approach for optimizing BLS education in undergraduate nursing, although multicenter studies with longer follow-up are needed to confirm generalizability and long-term retention.

## Introduction

Out-of-hospital cardiac arrest is a time-critical emergency in which prompt, high-quality basic life support (BLS) forms the pivotal first link in the chain of survival (1). For nursing students, BLS proficiency is both a core clinical competency and a cornerstone of professional identity formation (2); improving bystander cardiopulmonary resuscitation (CPR) among future healthcare professionals is accordingly regarded as a key global strategy to strengthen this chain (1). Yet nursing students continue to struggle with BLS acquisition and retention. Even after formal training, recurrent deficiencies persist, including insufficient compression depth, inefficient ventilation, and suboptimal use of the automated external defibrillator (3, 4); more concerningly, both knowledge and psychomotor skills decay rapidly, often within months (5, 6). This persistent gap between knowing and doing, together with poor long-term retention, exposes the limitations of prevailing BLS training paradigms in fostering deep learning and durable skill transfer.

Cultivating and assessing this full competency continuum requires a coherent theoretical framework. Miller’s pyramid, widely adopted in medical education, stratifies clinical competence into four ascending levels: knows, knows how, shows how, and does (7), and has profoundly shaped competency-based medical education by emphasizing level-specific teaching and assessment (8). Subsequent refinements have extended its apex: internalization of professional values into professional identity (is) has been proposed as a higher aspiration than does alone (2), while entrustment-based decision-making has been advanced as the operational core of does, reflecting supervisors’ judgment of the independence with which a learner can perform a given task (7). These additions reposition Miller’s pyramid from an assessment tool into a theoretical blueprint spanning knowledge transmission, behavioral internalization, and clinical entrustment (9). Consistent with this progression, BLS instruction for nursing students has shifted from teacher-centered lectures and massed practice, approaches of limited efficacy for long-term retention (5), toward technology-enhanced modalities. Online and blended learning sustain instructional continuity and improve theoretical knowledge (10, 11); high-fidelity simulation enhances student confidence in performing BLS (12); extended reality provides immersive environments supporting psychomotor skill acquisition (13, 14); and smartphone-based serious games offer novel means for autonomous knowledge consolidation (15). Comparative evidence, however, is inconsistent: self-directed learning has yielded skill performance comparable to traditional instruction (16); virtual reality has shown no clear superiority over conventional simulation, despite greater cognitive engagement (13); and blended learning integrating online and in-person components may deliver superior overall CPR quality (17). Such heterogeneity suggests that technological innovation, in the absence of a coherent pedagogical framework, may fall short of its full potential.

Despite abundant research on BLS teaching innovations, most studies compare isolated technologies or media without a unifying theoretical framework, leaving interventions to target only fragments of competence (8). Miller’s pyramid offers a means to bridge this gap, spanning knows and knows how via knowledge transmission, shows how through simulation, and does and entrustment, within authentic clinical contexts (7). However, how this framework can be operationalized within integrated instructional systems such as HeartCode BLS, which combines online theoretical learning, simulation practice, and automated feedback, to systematically enhance nursing students’ multilevel competence remains insufficiently examined. Existing studies have largely assessed single methods in isolation, providing inadequate evidence on whether a theoretically grounded, integrated model can concurrently and durably improve theoretical performance, skill execution, and long-term clinical application; its effect on learner satisfaction likewise warrants further investigation. We therefore undertook a randomized controlled trial comparing a HeartCode BLS instructional model informed by Miller’s pyramid with conventional traditional instruction in BLS training for nursing students, evaluating differences in immediate knowledge and skill acquisition, CPR quality, long-term integrated clinical application, and learner satisfaction, to provide a theoretically grounded, empirically validated approach for optimizing BLS education in undergraduate nursing.

## Subjects

This study is a randomized controlled trial. Sample size calculation was based on the superiority test formula for comparing two independent samples:


$$n=\frac{2\times p\times \left(1-p\right)\times {\left(Z_{1-\alpha /2}+Z\,1_{1-\beta }\right)}^{2}}{\delta^{2}}$$

The test level was set at two-tailed α = 0.05, corresponding to Z_1–α__/2_ = 1.96; the power (1-β) was set at 80%, corresponding to Z_1–β_ = 0.842. Based on pre-experimental data and prior relevant studies, the estimated overall compliance rate for critical operational points in the control group was p_1_ = 0.65, while the target achievement rate in the observation group was

P_2_ = 0.85. The pooled proportion was calculated as *p* = (p1 + p2)/2 = 0.75. The superiority margin was defined as δ = p2 - p1 = 0.20.

Calculations indicate a theoretical sample size of approximately 92 cases per group. Considering potential intra-group correlation due to the small-group teaching model, a design effect (DEFF = 1.15) was applied for adjustment. Additionally, accounting for factors such as student rotation and absences during clinical practice, a projected attrition rate of 15% was incorporated to further refine the sample size:


$$\mathrm{Adjust}\,\mathrm{sample}\,\mathrm{size}=\frac{92\times 1.15}{1-0.15}\approx 125$$

To ensure statistical power, the study planned to enroll at least 110 nursing students per group. A total of 257 nursing students were assessed for eligibility; 21 were excluded (11 did not meet the inclusion criteria, 8 declined to participate, and 2 for other reasons), and 236 students were randomized (118 per group). During the 3-month follow-up, 7 students were lost to follow-up (4 in the observation group and 3 in the control group, owing to internship-schedule conflicts or sick leave), leaving 229 students (114 in the observation group and 115 in the control group) for the final analysis. The total sample size and intergroup distribution both met and slightly exceeded theoretical requirements. The actual dropout rate was 3% (below the projected value), indicating sufficient statistical power for this study.

**FIGURE 1:**
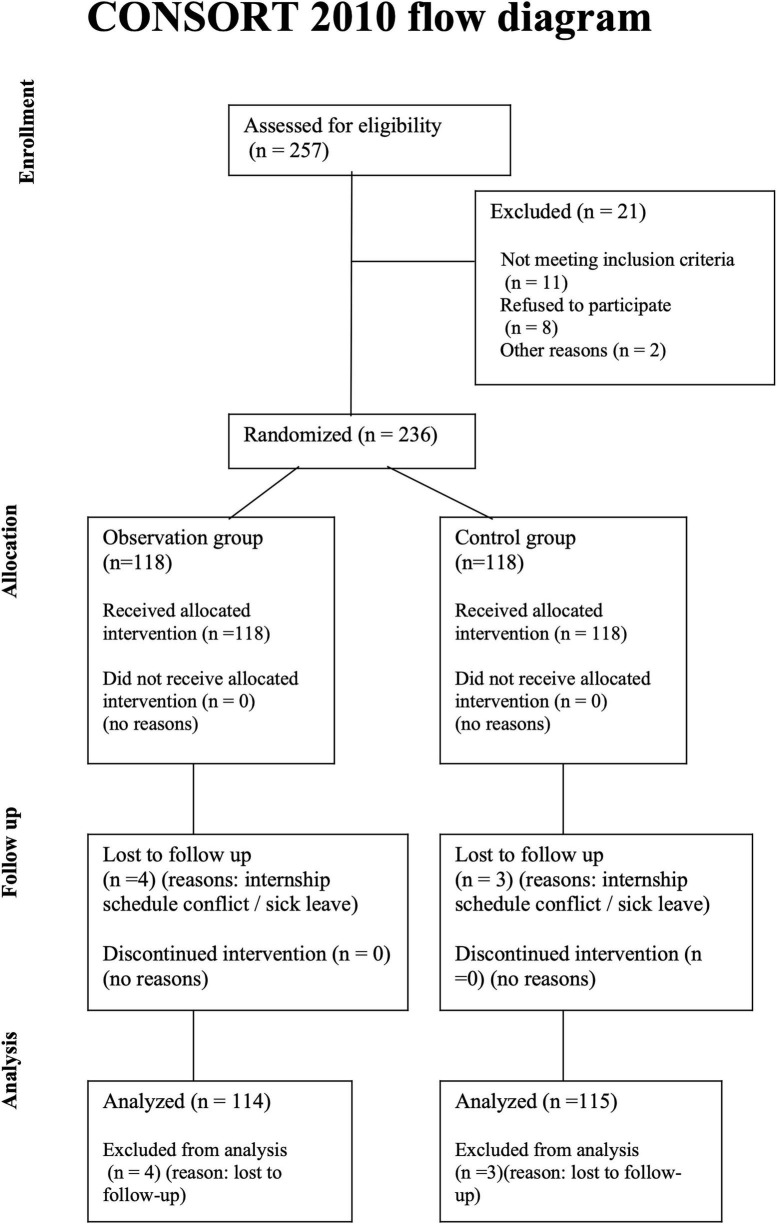
CONSORT 2010 flow diagram of participant enrolment, allocation, follow-up, and analysis. Of 257 nursing students assessed for eligibility, 21 were excluded (11 did not meet the inclusion criteria, 8 declined to participate, and 2 for other reasons), leaving 236 who were randomized (118 to the observation group and 118 to the control group). During the intervention period, 4 participants in the observation group and 3 in the control group were lost to follow-up, so that 114 and 115 participants, respectively, were included in the final analysis.

Inclusion criteria: (1) No prior systematic BLS training; (2) Voluntary participation with signed informed consent. Exclusion criteria: (1) Completion of Red Cross-certified emergency training courses; (2) Inability to complete full training and assessment due to leave, illness, etc. All participants were clinical-intern nursing students recruited from People’s Hospital of Quzhou (the Affiliated Quzhou Hospital of Wenzhou Medical University), Zhejiang, China. Eligible participants were randomly allocated to the control group or the observation group. The random allocation sequence was generated by an independent research assistant using a computer random-number generator, and allocation concealment was ensured using sequentially numbered, opaque, sealed envelopes that were opened in strict enrolment order by an on-site assistant only after baseline data collection had been completed. This trial was registered on the National Medical Research Registration and Filing Information System (registration number MR3325078976; 17 November 2025). The recruitment, allocation, follow-up, and analysis of participants are summarized in the CONSORT 2010 flow diagram (Figure 1). Comparisons of baseline characteristics between the two nursing student groups, including educational background, gender, age, and pre-training BLS-related theoretical and skill scores, revealed no statistically significant differences (*P* > 0.05), confirming comparability.

## Methods

### Training faculty formation

**FIGURE 2:**
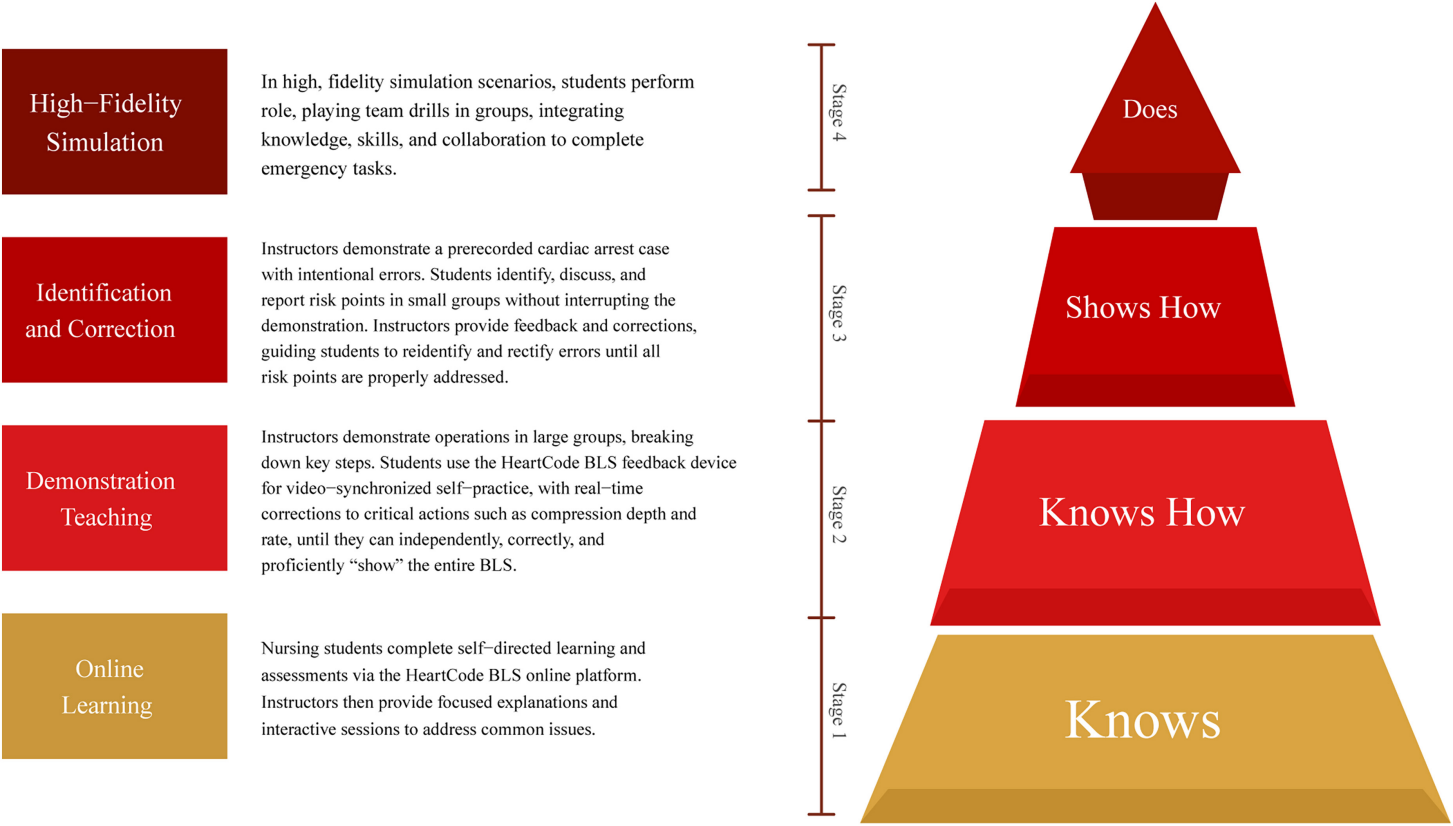
Miller’s pyramid-based HeartCode BLS teaching model.

The four-stage instructional workflow of the Miller’s Pyramid-based HeartCode BLS model is shown in Figure 2.

### Training faculty and student grouping

All training was delivered by eight American Heart Association (AHA)-certified BLS instructors, two of whom were dedicated solely to outcome assessment and did not participate in teaching. Before the study, all instructors completed a standardized 4-h calibration session covering the study protocol, the Miller’s pyramid framework, operation of the HeartCode BLS platform, and structured debriefing, in order to ensure instructional consistency. The same instructor team taught both groups, so as to minimize instructor-related bias, and the instructors were not informed of the study’s primary hypothesis regarding the superiority of the new model. Students were organized into learning teams of four to five, with an approximate student-to-instructor ratio of 14:1.

### Intervention duration and control-group teaching

Because the two instructional models differ structurally, the observation group included additional online learning, virtual-feedback practice, and high-fidelity team simulation, whereas the control group was delivered mainly through centralized lectures and group practice, the total instructional time could not be made exactly equal between groups, which is a common and largely unavoidable situation in educational-intervention research. The total instructional time differed by no more than 90 min between groups (observation group, 810 min; control group, 720 min). Apart from the instructional design itself, the two groups were kept consistent with respect to faculty, venue, manikin model, and assessment criteria, so as to maximize between-group comparability. The control group received conventional BLS training, defined as instructor-centered teaching delivered in a fixed sequence of theoretical lecture, instructor demonstration, and supervised group practice, without structured online pre-class learning, without device-based real-time feedback (relying instead on the instructor’s subjective and delayed feedback), and without high-fidelity team-based simulation. During the intervention period, the control group received no additional content from the new instructional model, and attendance and compliance were recorded for both groups by the study coordinators. The instructional structure, content, and time allocation of the two groups are summarized in Table 1.

**TABLE 1:** Instructional structure, content, and time allocation of the two groups.

Group	Instructional component (corresponding to Miller’s level)	Time (min)
Observation group	Online HeartCode BLS cognitive self-study and synchronous quiz (Knows/Knows How)	90
	Instructor-led interpretation of key knowledge points and interactive Q&A (Knows How)	90
	Large-group demonstration with stepwise breakdown of CPR (Shows How)	180
	QCPR virtual real-time-feedback skills practice (Shows How)	90
	Scenario-based risk-point identification training (Shows How/Does)	180
	High-fidelity team simulation and structured debriefing (Does/Trusted)	180
	Observation group subtotal	810
Control group	Centralized theoretical lecture with discussion (Knows/Knows How)	180
	Large-group instructor demonstration (Shows How)	180
	Small-group supervised practice (Shows How)	360
	Control group subtotal	720

### Standardization of the simulation activities

Two validated cardiac-arrest scenarios were used. Scenario A (witnessed in-hospital ventricular-fibrillation arrest) was used for observation-group training, and Scenario B (unwitnessed pulseless electrical activity) was used for the 3-month high-fidelity assessment of both groups. The two scenarios were judged to be of comparable complexity by two senior emergency-education experts who were not otherwise involved in the study. All simulations were conducted on the same high-fidelity manikin; each simulation was strictly limited to 10 min and was followed by a 15-min structured debriefing. Instructor prompts were fully scripted, and identical facilitation language was used for all student teams. The observation group completed all three mandatory HeartCode BLS modules: (1) the online cognitive course; (2) virtual-simulation skills practice (covering adult, child, and infant CPR and AED use); and (3) the skills test. These modules are also depicted in Figure 2.

### Feedback-enabled instructional strategy

The Anne QCPR real-time feedback system provided an automated, device-based dashboard (compression rate, depth, and recoil; ventilation; and defibrillation) without requiring instructor judgment, and feedback was delivered primarily at the individual level during skills practice. In addition, a 15-min structured debriefing was conducted after each simulation, in which the instructor guided students through the QCPR report and the video recording, focusing on team-based application of skills such as leadership, communication, and task allocation.

### Blinding

Owing to the nature of the instruction, the six teaching instructors could not be blinded, but they were unaware of the specific between-group comparisons (e.g., the achievement-rate thresholds and the content of the 3-month assessment). The two assessors were fully blinded: the two AHA-certified instructors who conducted the 1-week skills assessment and the 3-month simulation assessment did not participate in any teaching and remained unaware of group allocation throughout. The data analyst was also fully blinded; the statistician received a de-identified dataset labeled only as “Group A” and “Group B,” and the allocation was unmasked only after the primary analysis had been completed.

### Evaluation indicators

This study adopts the classic Kirkpatrick Four-Level Evaluation Model (18, 19) as its overall assessment framework. Proposed by Donald L. Kirkpatrick in 1959, this model is an internationally recognized method for evaluating training effectiveness. It assesses performance across four dimensions: Reaction, Learning, Behavior, and Results. The specific evaluation plan is designed as follows:

### Learning level (theoretical test)

Following training, both groups of trainees underwent a standardized closed-book theoretical examination scored out of 100 points to assess their mastery of core Basic Life Support knowledge.

### Behavioral level (skill assessment)

One week after training, two AHA-certified instructors conducted skill assessments to evaluate the proficiency of both groups of nursing students. Scoring utilized a rubric developed based on authoritative references, including the 2020 American Heart Scoring was performed using a rubric developed with reference to the 2020 American Heart Association Guidelines for Cardiopulmonary Resuscitation and Emergency Cardiovascular Care and related clinical practice standards (20). Simultaneously, the quality of CPR-related indicators will be observed, including the accuracy rates for compression depth, rate, recoil, and bag-valve-mask ventilation. According to guidelines, Correct compression depth is 5th cm, compression rate is 100s 5 the accuracy ratesminute with full chest recoil, and bag-valve-mask ventilation delivers 500bagh mL tidal volume per breath with a 1-s ventilation duration. Compliance with these standards was judged as correct (21), with data automatically recorded by the Anne QCPR real-time feedback system.

### Outcomes layer (comprehensive application skills simulation assessment)

A high-fidelity scenario simulation assessment was conducted 3 months post-training, scored out of 100 points. Assessment criteria followed the 2020 American Heart Association Guidelines for Cardiopulmonary Resuscitation and Emergency Cardiovascular Care, evaluating application skills across three dimensions: Emergency Judgment and Decision-Making (30%), Emergency Procedure Execution (40%), and Team Communication and Collaboration (30%), encompassing 15 content areas.

### Response layer (training satisfaction evaluation)

A teaching satisfaction questionnaire was developed with reference to previous medical education studies (22) and adapted to the objectives of the present training program. The questionnaire comprised five items: enhancing learning interest, improving clinical critical thinking skills, boosting clinical emergency response capabilities, strengthening the ability to address real clinical problems, and overall satisfaction with the training model. Responses were rated on a 5-point Likert scale ranging from 1 (strongly disagree) to 5 (strongly agree), with higher scores indicating greater satisfaction. In the present study, the questionnaire demonstrated good internal consistency, with a Cronbach’s alpha coefficient of 0.838. After completion of the training, the satisfaction survey was administered to both groups via Questionnaire Star, and the valid response rate was 100%.

### Statistical analysis

Data processing and statistical analyses were performed using SPSS AU statistical software. Categorical variables are presented as frequencies and percentages [n (%)], and between-group comparisons were conducted using the chi-square test. Continuous variables are expressed as the mean ± standard deviation (SD). Normality was assessed before analysis. For normally distributed continuous variables, between-group comparisons were performed using the independent-samples *t*-test. For non-normally distributed continuous variables, the Mann-Whitney U test was used. For repeated-measures data assessing the magnitude of improvement before and after training, repeated-measures analysis of variance (ANOVA) with Bonferroni correction was applied for multiple comparisons. Effect sizes were evaluated using generalized eta squared (ges), with ges ≥ 0.01 indicating a small effect, ges ≥ 0.06 indicating a medium effect, and ges ≥ 0.14 indicating a large effect. A two-sided *P* < 0.05 was considered statistically significant. Learning teams of four to five students were formed by computer-generated random assignment rather than self-selection, and team membership remained fixed from the initial training through the 3-month follow-up assessment. For the primary individual-level outcomes (theoretical knowledge, individual skill performance, and satisfaction; *n* = 229), the individual student was the unit of analysis, and the potential clustering arising from the small-group teaching format was accounted for in the sample-size calculation by applying a design effect (DEFF = 1.15). For the 3-month high-fidelity simulation, which assessed integrated team performance, the team was the unit of analysis (23 teams per group).

## Results

### Baseline comparability of the two groups

Before training, the two groups of nursing students were compared across educational background, gender, age, and baseline BLS-related theoretical and skill scores. No statistically significant differences were identified in any of these variables (all *P* > 0.05), confirming that the two groups were well-matched at baseline and that subsequent between-group comparisons were methodologically sound (Tables 2, 3). The enrolled students represented the typical composition of clinical-intern nursing students, comprising 19 from technical secondary schools, 166 from junior colleges, and 44 undergraduates; these categories denote the students’ pre-service nursing qualification level, and their distribution did not differ significantly between the two groups (Table 2).

**TABLE 2:** Comparison of educational background, gender, and age between two groups of nursing students.

Characteristic	Observation group (*n* = 114)	Control group (*n* = 115)	Statistical value	*P*-value
Educational background, n (%)			χ^2^ = 0.508	0.776
Undergraduate	24 (21.1)	20 (17.4)		
Junior college	81 (71.1)	85 (73.9)		
Technical secondary school	9 (7.9)	10 (8.7)		
Gender, n (%)			χ^2^ = 0.217 *t* = 0.372	0.641 0.710
Female	93 (81.6)	91 (79.1)		
Male	21 (18.4)	24 (20.9)		
Age (years), mean ± SD	20.04 ± 1.25	19.97 ± 1.23		

**TABLE 3:** Comparison of baseline BLS-related theory and skill scores between two groups of nursing students before training.

Item	Observation group (*n* = 114)	Control group (*n* = 115)	*T*-value	*P*-value
Knowledge test score	62.72 ± 9.11	62.59 ± 8.89	0.108	0.914
Skills score				
Total CPR score	61.28 ± 3.97	61.80 ± 5.92	–0.774	0.440
Defibrillation score	45.86 ± 4.19	45.18 ± 4.58	1.175	0.241
Skill total score	107.15 ± 6.02	106.97 ± 7.94	0.178	0.859

### Comparison of post-training theoretical and skill scores

Following the training intervention, repeated-measures ANOVA was used to evaluate the group-by-time interaction effects on theoretical knowledge, CPR performance, and defibrillation scores. In all three domains, statistically significant interaction effects were observed (all *P* < 0.001), demonstrating that Miller’s Pyramid-based HeartCode BLS teaching model produced substantially greater learning gains than the traditional approach. The observation group achieved markedly higher post-training scores than the control group across theoretical knowledge (74.29 ± 9.94 vs. 68.81 ± 9.33), CPR skills (85.92 ± 8.67 vs. 79.80 ± 7.56), and defibrillation skills (85.48 ± 8.63 vs. 78.86 ± 5.77).

Notably, the generalized eta-squared (Ges) values for CPR (0.252) and defibrillation (0.292) indicated large effect sizes, substantially exceeding the medium effect observed for theoretical knowledge (0.092). This pattern suggests that the structured, technology-enhanced instructional model exerted a particularly pronounced influence on psychomotor skill development, where greater room for improvement existed compared to cognitive knowledge acquisition (Table 4).

**TABLE 4:** Comparison of theoretical and individual skill scores between two groups of nursing students after training.

Assessment Indicators	Group	N	Pre-training	Post-training	Main effect of time		Main effect of group		Group × time interaction		
					*F*	*P*	*F*	*P*	*F*	*P*	Ges
Theoretical score	Obs	114	62.68 ± 9.06	74.29 ± 9.94	1878.024	<0.001	25.051	<0.001	410.696	<0.001	0.092
	Ctrl	115	62.59 ± 8.89	68.81 ± 9.33							
CPR score	Obs	114	61.28 ± 3.97	85.92 ± 8.67	6689.157	<0.001	77.400	<0.001	462.171	<0.001	0.252
	Ctrl	115	61.80 ± 5.92	79.80 ± 7.56							
Defibrillation score	Obs	114	45.86 ± 4.19	85.48 ± 8.63	8726.235	<0.001	200.300	<0.001	218.581	<0.001	0.292
	Ctrl	115	45.18 ± 4.58	78.86 ± 5.77							

### Comparison of CPR quality achievement rates

Post-training assessment of CPR quality indicators revealed that the observation group achieved significantly higher compliance rates across all four key metrics—compression depth, compression rate, chest recoil, and bag-valve-mask ventilation—compared with the control group (all *P* < 0.001). Mann-Whitney U tests confirmed these differences, with effect sizes (r) ranging from 0.515 to 0.642, uniformly exceeding the threshold for a large effect (r > 0.5). Among these, bag-valve-mask ventilation showed the most pronounced intergroup difference (median: 80.00% vs. 55.00%; *r* = 0.642), suggesting that the real-time feedback mechanism embedded in the HeartCode BLS system was particularly effective in refining this technically challenging skill component.

**FIGURE 3:**
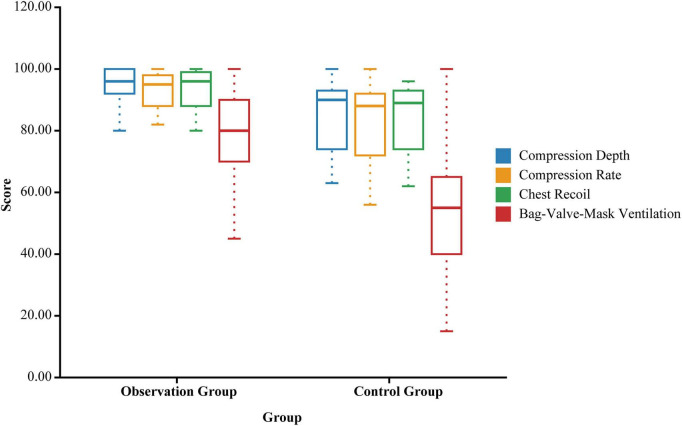
Comparison of performance on key BLS quality indicators between the two groups of nursing students.

Furthermore, analysis of total compression interruption time (Table 5) demonstrated that a significantly greater proportion of nursing students in the observation group maintained interruptions below 30 s (57.0% vs. 25.2%; χ^2^ = 25.347, *P* < 0.001, Cramér’s *V* = 0.333), reflecting superior compression continuity and overall procedural fluency under the new teaching model (Figure 3 and Tables 5, 6).

**TABLE 5:** Comparison of total compression interruption time between two groups of nursing students.

Metric	Observation group (*n* = 114)	Control group (*n* = 115)	Statistical value	*P*-value	Cramér’s V
Interruption time category, n (%)			χ^2^ = 25.347	< 0.001	0.333
<30 s	65 (57.0)	29 (25.2)			
30–60 s	40 (35.1)	62 (53.9)			
>60 s	9 (7.9)	24 (20.9)			

**TABLE 6:** Comparison of quality achievement rates at key operational points between two groups of nursing students after training.

Indicator	Observation group (*n* = 114)	Control group (*n* = 115)	Mann-Whitney U	*Z*-value	*P*-value	*r*
Compression depth	96.00 (91.8, 100.0)	90.00 (74.0, 93.0)	2546.00	–8.020	<0.001	0.529
Compression rate	95.00 (88.0, 98.0)	88.00 (72.0, 92.0)	2652.50	–7.796	<0.001	0.515
Chest recoil	96.00 (88.0, 99.0)	89.00 (74.0, 93.0)	2410.00	–8.283	<0.001	0.547
Bag-valve-mask ventilation	80.00 (70.0, 90.0)	55.00 (40.0, 65.0)	1696.50	–9.721	<0.001	0.642

### Comparison of training satisfaction

Across all five satisfaction dimensions and the composite total score, the observation group reported significantly higher ratings than the control group (all *P* < 0.001). Effect sizes (r) ranged from 0.677 to 0.863, consistently indicating large practical effects. The item “improved clinical emergency response ability” exhibited the largest intergroup difference (median: 5.00 vs. 2.00; *r* = 0.863), suggesting that the immersive, scenario-based training experience most powerfully enhanced students’ perceived readiness for emergency clinical situations. The total evaluation score further confirmed robust overall satisfaction in the observation group (median: 25.00 vs. 16.00; *r* = 0.845) (Table 7).

**TABLE 7:** Comparison of training satisfaction between two groups of nursing students.

Item	Observation group (*n* = 114)	Control group (*n* = 115)	Mann-Whitney U	*Z*-value	*P*-value	r
Improved learning interest	5.00 (5.0, 5.0)	3.00 (3.0, 4.0)	1675.50	–10.363	<0.001	0.685
Improved clinical critical thinking ability	5.00 (5.0, 5.0)	4.00 (3.0, 4.0)	1763.00	–10.243	<0.001	0.677
Improved clinical emergency response ability	5.00 (5.0, 5.0)	2.00 (1.0, 3.0)	255.00	–13.055	<0.001	0.863
Improved ability to solve practical clinical problems	5.00 (4.8, 5.0)	3.00 (2.0, 4.0)	907.00	–11.771	<0.001	0.778
Satisfaction with the training mode	5.00 (4.8, 5.0)	3.00 (2.0, 4.0)	897.00	–11.869	<0.001	0.784
Total score of evaluation	25.00 (24.0, 25.0)	16.00 (14.0, 18.0)	296.50	–12.789	<0.001	0.845

### Comparison of clinical comprehensive application competency simulation scores

Three months after the completion of training, high-fidelity simulation assessments were conducted at the team level (23 teams per group). The observation group significantly outperformed the control group in all three competency dimensions, emergency judgment and decision-making, emergency operation and execution, and team communication and collaboration, as well as in the total composite score (all *P* < 0.001). The effect sizes (r) ranged from 0.314 to 0.381, indicating moderate-to-large effects and meaningful intergroup differences.

**FIGURE 4:**
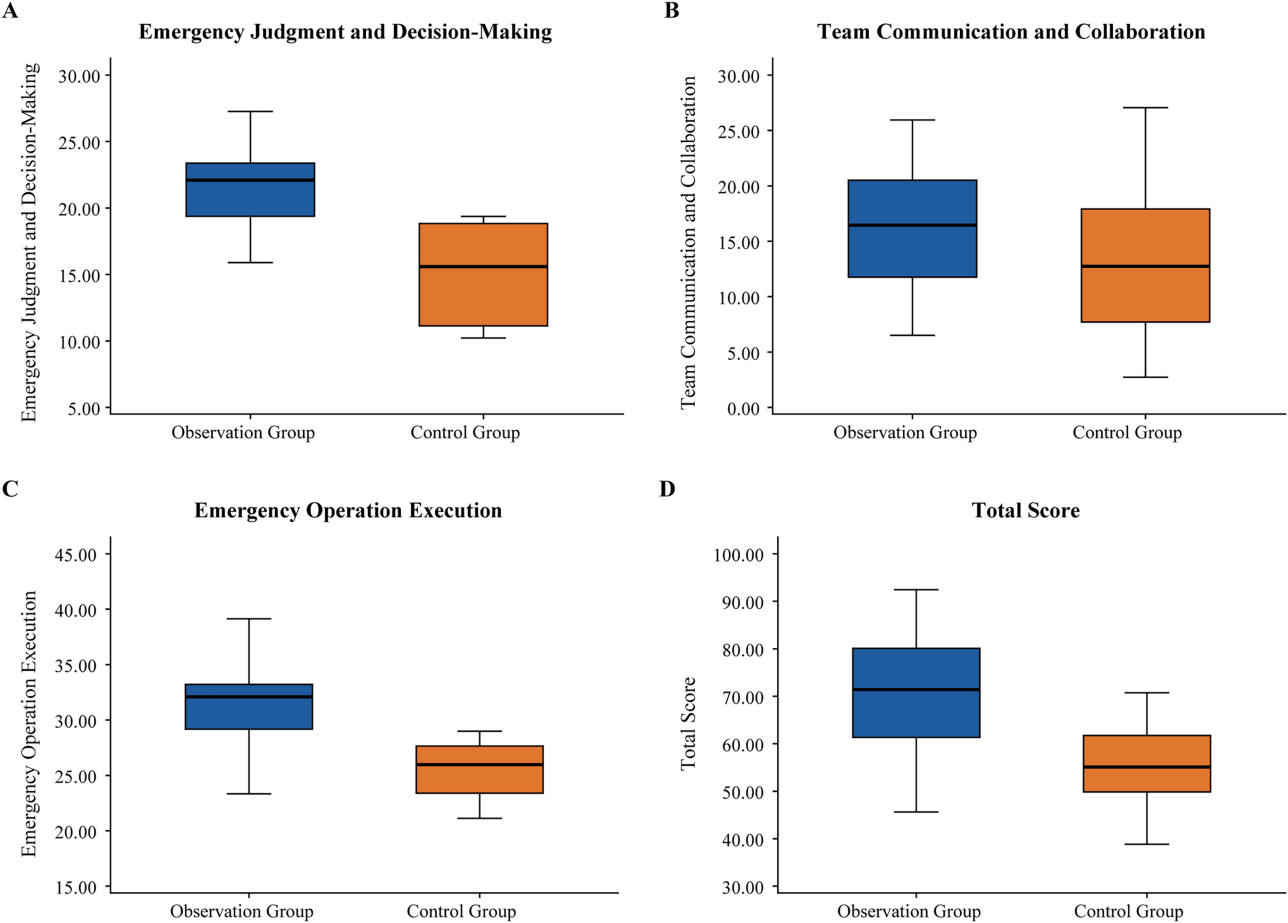
Comparison of clinical comprehensive application ability simulation test scores between the observation and control groups 3 months after training. Box plots illustrate the distribution of scores in four domains of the high-fidelity simulation assessment: **(A)** emergency judgment and decision-making, **(B)** team communication and collaboration, **(C)** emergency operation execution, and **(D)** total score. Across all four domains, the observation group achieved higher scores than the control group.

The largest effect was observed in the total score dimension (median: 68.48 vs. 45.34; *r* = 0.381), while team communication and collaboration also showed a notable difference (median: 16.50 vs. 4.50; *r* = 0.359). These findings indicate that the Miller’s Pyramid-based model not only enhanced individual skill proficiency but also effectively cultivated higher-order clinical competencies such as situational decision-making, adaptive resource allocation, and collaborative leadership within team-based emergency scenarios (Figures 4A–D and Table 8).

**TABLE 8:** Comparison of clinical comprehensive application ability simulation test scores between two groups of nursing students after training.

Item	Observation group (*n* = 23)	Control group (*n* = 23)	Mann-Whitney U	*Z*-value	*P*-value	*r*
Emergency judgment and decision-making	22.13 (19.5, 23.7)	15.50 (11.0, 19.0)	40.50	–4.925	<0.001	0.325
Emergency operation and execution	32.07 (28.7, 33.0)	26.02 (23.2, 27.7)	48.50	–4.746	<0.001	0.314
Team communication and collaboration	16.50 (10.5, 21.0)	4.50 (4.0, 6.5)	18.00	–5.430	<0.001	0.359
Total score	68.48 (60.4, 73.3)	45.34 (40.5, 50.4)	2.00	–5.767	<0.001	0.381

## Discussion

The present study employed a randomized controlled trial to systematically compare the effectiveness of the Miller’-pyramid-based HeartCode BLS instructional model against conventional instruction for BLS training in nursing students. The observation group significantly outperformed the control group across multiple outcome domains, including theoretical examination scores, CPR skill performance, defibrillation skill performance, training satisfaction, and simulated integrated clinical performance assessed 3 months after training. Collectively, these findings indicate that the HeartCode BLS model confers demonstrable advantages not only in knowledge acquisition and psychomotor skill development but also in fostering sustained integrative clinical competence and a more positive learning experience. By coherently integrating digitally delivered self-paced learning with high-fidelity simulation feedback, the model aligns closely with Miller’s pyramid framework, spanning the hierarchical continuum from “Knows” to “Does,” and ultimately to the “Trusted” level, thereby providing novel empirical evidence to inform the optimization of BLS training in nursing education.

The observed gains in theoretical knowledge constitute one of the principal findings of this study. Miller’s pyramid delineates four hierarchical levels of clinical competence: knowledge (Knows), application of knowledge (Knows How), behavioral demonstration (Shows How), and authentic practice (Does). Ten Cate and colleagues have argued for the addition of a fifth, apex tier termed “Trusted,” which represents educators’ confidence in trainees to independently assume clinical responsibility (7). The HeartCode BLS platform scaffolds self-paced acquisition of core BLS knowledge through interactive digital modules, reinforced by scenario-based application exercises, thereby effectively strengthening the “Knows” and “Knows How” tiers. In a parallel vein, Prabu Kumar et al. demonstrated that e-learning modules designed according to Bloom’s taxonomy and Miller’s pyramid significantly enhance medical students’ performance across cognitive domains encompassing remembering, understanding, applying, and analyzing (23). The knowledge gains reported in the current study are consistent with, and appear more pronounced than, those of earlier work, a discrepancy plausibly attributable to the embedded immediate-assessment and targeted-feedback mechanisms within the HeartCode platform, which encourage active cognitive engagement rather than passive reception of information. In a related line of inquiry, Ranjbar et al. reported that nursing students trained with spaced e-learning for BLS demonstrated significantly superior knowledge retention compared with those undergoing a single, massed learning session (24). Although the HeartCode model does not explicitly prescribe a spaced schedule, its modular architecture and repeatable access effectively permit learners to calibrate review cadence to their own mastery, thereby promoting memory consolidation. Flipped-classroom approaches have likewise demonstrated efficacy in BLS training; for example, Natarajan et al. reported significantly improved CPR knowledge scores among nursing students, together with a positive association between knowledge and skill performance (25). The theoretical advantage observed in the present study may therefore be partly ascribed to the integration of active-learning elements within HeartCode, wherein a self-paced, feedback-rich environment fosters deeper understanding and internalization of knowledge to a greater degree than traditional didactic instruction.

With respect to psychomotor performance, the observation group achieved significantly higher CPR and defibrillation skill scores than the control group, and also exhibited a higher proportion of CPR-quality compliance. These results stand in striking contrast to the well-documented challenges of skill retention reported in the literature. Charlier et al. observed that nursing students demonstrated significant deterioration across several critical BLS parameters, most notably ventilation volume, compression depth, and compression rate, as early as 4 months post-training (6). An integrative review by Dick-Smith et al. similarly underscored that existing instructional approaches cannot reliably secure both the acquisition and long-term retention of BLS skills, and advocated the incorporation of objective feedback technologies to enhance psychomotor learning (26). The real-time feedback system embedded within HeartCode BLS is purposefully engineered to address this gap: as learners perform chest compressions and ventilations on the manikin, the system delivers immediate visual feedback on depth, rate, and chest recoil, enabling on-the-fly self-correction. Oermann et al. demonstrated that low-dose, high-frequency Resuscitation Quality Improvement (RQI) programs, delivering quarterly CPR practice sessions combined with real-time feedback, effectively maintain and even enhance students’ compression and ventilation skills (27, 28). The skill gains observed in the present study closely parallel these mechanisms. Ghaderi et al. compared real-time feedback with *post-hoc* video debriefing in BLS training and found that, although both modalities were effective, real-time feedback conferred a distinct advantage for on-the-spot correction of technique (29). The contribution of high-fidelity simulation to skill acquisition has also received broad recognition; Rushton et al. reported that high-fidelity simulation significantly enhanced learners’ confidence in BLS performance (12), whereas a randomized trial by Mather et al. found no statistically significant improvement in CPR performance metrics attributable to high-fidelity environments (30), suggesting that skill improvement may depend more on feedback quality than on environmental realism per se. Bodur et al. and Fijaèko et al. further validated the utility of virtual reality and serious games in CPR training, with the VR group in the former study outperforming conventional simulation across several key compression parameters (13, 15). The substantial improvement in defibrillation skill observed in the current study is most plausibly attributable to the structured, component-wise training afforded by the HeartCode platform, spanning electrode-pad placement, energy selection, and safety verification, each component being subject to repeated practice and immediate assessment, a level of granularity frequently compromised by time constraints in conventional instruction.

Training satisfaction represents a critical dimension in evaluating the feasibility of educational innovations. In the present study, the observation group recorded significantly higher scores than the control group across all satisfaction subscales, in line with multiple prior investigations of technology-enhanced BLS training. Khaledi et al. contrasted gamification with role-play instruction and demonstrated that the gamification group exhibited significantly greater CPR self-efficacy than those receiving traditional didactic instruction (31). HeartCode BLS incorporates gamified elements, such as level progression, point accumulation, and leaderboards, that effectively stimulate learners’ intrinsic motivation. Both Peinado-Molina et al. and Demirtas et al. have reported significant post-simulation improvements in self-efficacy and satisfaction (32, 33). Torbergsen et al. examined the associations among autonomous motivation, perceived teacher goals, and learning outcomes within a flipped-classroom learning design, and reported that students’ perceptions of teacher goals and their learning effort were significantly positively correlated with learning outcomes (34). In the present study, the HeartCode model affords learners substantially greater learning autonomy while concurrently guiding them through clearly articulated stage-specific objectives aligned with successive levels of Miller’s pyramid; such a learner-centered design is conducive to enhanced satisfaction and engagement.

Of particular note, at the 3-month follow-up, the observation group continued to demonstrate significantly higher scores on the simulated integrated clinical performance assessment, indicating that the HeartCode model not only accelerates short-term skill acquisition but also improves mid-term skill retention. Skill retention has long constituted a persistent challenge in BLS education. Soares et al. demonstrated that the combined application of spacing and testing effects significantly improved 3-month retention of both basic and advanced life support skills, whereas learners receiving a single simulation session exhibited substantial skill decay (5). The sustained advantage observed in the present study may derive from two complementary mechanisms: first, the HeartCode platform enables repeated engagement with key skill modules throughout the training period, thereby fostering more durable motor memory; second, the model deliberately integrates knowledge, skills, and clinical decision-making within unified scenarios, and such holistic training promotes deeper understanding and more reliable memory retrieval. A systematic review by Daneshfar et al. reported that high-fidelity simulation and structured scenario-based interventions effectively narrow the theory-practice gap and enhance clinical decision-making, judgment, and confidence (35). Liu et al., applying the Kirkpatrick model to evaluate an “online–simulation–bedside” three-step instructional approach, found that it significantly improved nursing interns’ teamwork capacity, clinical reasoning, and skill-related self-confidence, with skill assessment scores reaching a level of excellence (36). The integrative-performance advantage observed in the observation group of the present study is therefore best interpreted as reflecting the nascent emergence of the apex “Trusted” competency of Miller’s pyramid: learners not only mastered discrete technical maneuvers but were also able to coordinate multiple procedural components within simulated clinical contexts, assessing responsiveness, summoning help, initiating CPR, deploying an AED, thereby exhibiting competencies in decision-making and adaptive performance. Ten Cate et al. have emphasized that the “Trusted” tier requires learners to possess the experience and judgment necessary to manage unanticipated challenges, extending well beyond procedural proficiency alone (7). By incorporating diverse scenarios of progressively increasing complexity, HeartCode BLS affords learners meaningful opportunities to cultivate such integrative competence. It should nonetheless be emphasized that these outcomes were obtained under controlled, simulated conditions rather than in authentic clinical practice. The present findings should therefore be interpreted as reflecting an emergent, simulation-based form of clinical transfer rather than verified independent clinical performance; accordingly, terms such as “Trusted,” clinical entrustment, and independent clinical competence are used here in a measured sense to denote progress toward, rather than full attainment of, the apex of Miller’s pyramid.

From the perspectives of clinical relevance and implementation, the Miller’s-pyramid-based HeartCode BLS model exhibits clear advantages in standardization, reproducibility, and cost-effectiveness. Traditional BLS instruction depends heavily on instructor expertise and on-site equipment, rendering educational quality and outcomes vulnerable to variability in faculty proficiency and facility resources. Koyuncu et al. reported that autonomous practice with low-cost pillow manikins significantly improved psychomotor performance in BLS, although such tools inherently lack real-time feedback functionality (37). The HeartCode platform, by contrast, achieves a uniformly standardized training experience through digital delivery, ensuring that each learner receives equivalent knowledge exposition and skill demonstrations while its feedback mechanisms remain unaffected by inter-instructor variability, thereby securing comparability of training outcomes. The model is particularly well-suited to large-scale deployment, for instance, as a substitute for conventional group-based instruction in nursing schools or in the orientation of newly hired nurses, where it can meaningfully alleviate faculty workload and enhance training efficiency. A systematic review by Alharbi et al. indicated that simulation-based learning (SBL) significantly improves nursing students’ knowledge and skills, although evidence for long-term retention remains limited, underscoring the need for more systematic instructional design (38). HeartCode’s modular architecture provides educational administrators with flexibility to configure hybrid (online-offline) content, and supports extension in line with contemporary interprofessional education trends. Fenzi et al. demonstrated that medical and nursing students participating in interprofessional CPR training using the Self-Learning Methodology in Simulated Environments (MAES) exhibited gains in teamwork, communication, and both technical and non-technical competencies (39). Incorporating HeartCode into interprofessional curricula, or deploying it as a preparatory component, may further optimize resource utilization. Tseng et al. demonstrated that integrating clinical simulation scenarios with information-technology-enhanced instruction significantly improved nursing students’ performance on objective structured clinical examinations (OSCEs) and their situational awareness in the management of acute myocardial infarction, BLS, and subdural hemorrhage (40). The successful experience of the present study suggests that framing Miller’s pyramid as a guiding structure, incorporating HeartCode as a core component of the nursing BLS curriculum, and complementing it with subsequent bedside practice and periodic refresher training, offers a promising pathway for constructing a coherent competency development pipeline extending from foundational cognition to clinical competence.

Regarding practical feasibility, the model can be implemented within the resources already available in most teaching hospitals. The total instructional time was comparable between the two groups (a difference of no more than 90 min; Table 1), and the principal resources required, QCPR feedback-enabled manikins and AHA-certified instructors, form part of the standard configuration of teaching hospitals, so that the additional cost is largely attributable to the licensing of the digital platform rather than to new equipment. No major barriers to implementation were encountered beyond occasional scheduling conflicts, which were resolved by arranging two make-up sessions. These observations suggest that the model is practical and scalable for undergraduate nursing programs and for the orientation of newly recruited nurses, although adequate technical support and routine device maintenance should be anticipated when the model is scaled up.

Notwithstanding these encouraging findings, several limitations warrant acknowledgment. First, the study employed a single-center design, and the sample was drawn exclusively from one nursing institution, which may constrain the external generalizability of the findings. Given institutional variability in educational resources, faculty expertise, and baseline student characteristics, the effectiveness of HeartCode requires corroboration in multicenter settings. Second, although integrated clinical performance was assessed at 3 months, longer-term follow-up (for example, 6 or 12 months) was not conducted. Oermann et al. demonstrated that quarterly low-dose, high-frequency CPR practice effectively sustains skill levels, but longitudinal evidence on retention beyond 6 months in the absence of feedback remains scarce (28). Long-term retention constitutes a critical indicator of any training program’s practical utility, and the available evidence remains insufficient. Third, all skill evaluations were conducted in a simulated environment, and learners’ actual performance in authentic clinical settings was not assessed. Although simulation-based assessment correlates positively with clinical competence to some degree, it cannot fully capture the stress responses and team-coordination dynamics inherent to real clinical encounters. Fourth, instructor-related variables were not fully controlled; although the HeartCode model attenuates variability in demonstration-based instruction, differences in instructors’ engagement during question-answer and facilitation segments between the two groups may have introduced confounding. Moreover, the observation group’s reliance on the HeartCode platform implies that technical disruptions, such as network outages or device-calibration issues, could potentially interfere with the training process; although no such incidents were reported in the present study, adequate technical support must be considered in future scale-up. Additionally, the study was not blinded, such that assessors’ awareness of group allocation may have introduced measurement bias; concurrently, students exposed to a novel instructional format may have manifested a Hawthorne effect, with heightened engagement and performance.

Future investigations may extend in several directions. First, multicenter studies with larger samples and prolonged follow-up are warranted to evaluate the impact of the HeartCode model on skill retention at 6 and 12 months, and to collect data on CPR performance in authentic clinical contexts, such as resuscitation quality during in-hospital cardiac arrest events. Second, the interaction between diverse feedback modalities and HeartCode merits further investigation. Ghaderi et al. compared real-time feedback with video-based debriefing for BLS skill instruction, finding both to be effective but complementary in focus (29). Future designs might integrate both feedback modalities within the HeartCode platform, or deploy them sequentially across distinct training phases, so as to maximize learning outcomes. Third, against the backdrop of accelerating advances in artificial intelligence, a systematic review by Yoon et al. indicated that generative AI holds promise for enhancing cognitive and affective outcomes in nursing education, while also presenting risks such as factual inaccuracy and clinical judgment failures, thereby advocating an “expert-in-the-loop” framework for integration (41). Embedding AI-driven virtual patients or intelligent tutoring systems within HeartCode could enable dynamic calibration of task difficulty and feedback content in response to learner performance, further personalizing the learning trajectory. Fourth, the maturation of virtual reality (VR) technology has established an immersive environment for BLS training; Bodur et al. reported that, although VR-based training markedly improved knowledge acquisition, gains in psychomotor skill were comparatively limited (13). Future research could therefore compare screen-based interactive simulation (as in standard HeartCode) with a VR-enabled HeartCode variant to examine differences in skill acquisition and retention, and to identify optimal configurations of combined technologies. Fifth, cost-effectiveness analyses should be incorporated to compare the HeartCode model with conventional instruction in terms of total costs, encompassing equipment investment, faculty time, and student workload, and associated outcomes, thereby providing economic evidence to inform institutional decision-making. Sixth, hybrid instructional designs warrant comparative evaluation, for instance, flipped-classroom instruction combined with HeartCode versus flipped-classroom instruction alone, or integration of HeartCode with high-fidelity simulation. Natarajan et al. have reported that the flipped classroom enhances CPR knowledge and skills (25), yet its integration with HeartCode’s digital feedback may yield synergistic effects. Finally, the Miller’s pyramid framework adopted here applies not only to BLS training but also to more complex skill domains, including advanced life support, airway management, and trauma resuscitation; subsequent investigations could thus examine its transferability across curricula. Through iterative refinement of instructional design and technological support, it may be possible to establish a more efficient, reproducible, and competency-oriented paradigm for nursing emergency education.

## Conclusion

The Miller’s Pyramid-based HeartCode BLS teaching model significantly improved theoretical knowledge, psychomotor performance, CPR quality, learner satisfaction, and 3-month comprehensive simulation performance among undergraduate nursing students compared with traditional instruction. These findings suggest that integrating competency-based pedagogy with digital learning, real-time feedback, and scenario simulation can strengthen both immediate skill acquisition and the transfer of BLS competence into higher-order clinical application. This model may represent a practical and scalable strategy for modernizing undergraduate nursing emergency training.

## Data Availability

The raw data supporting the conclusions of this article will be made available by the authors, without undue reservation.
